# Examining activity-friendly neighborhoods in the Norwegian context: green space and walkability in relation to physical activity and the moderating role of perceived safety

**DOI:** 10.1186/s12889-023-15170-4

**Published:** 2023-02-06

**Authors:** Vilde Juul, Emma Charlott Andersson Nordbø

**Affiliations:** Department of Public Health Science, Faculty of Landscape and Society, Norwegian University of Life Sciences, PO Box 5003, NO-1432 Ås, Norway

**Keywords:** Physical activity, Built environment, Perceived safety, Green space, Walkability.

## Abstract

**Background:**

Despite the well-known health benefits of regular physical activity, inactivity remains a major public health concern. Understanding how the built environment can encourage physical activity is therefore important to inform current policy strategies for creating activity-friendly neighborhoods. This study aimed to examine whether neighborhood walkability and greenness were associated with physical activity, and if perceived safety moderated any such relations, among adult citizens in Norway.

**Methods:**

This cross-sectional study included a sample of 5670 adults aged ≥ 18 years living in urban areas of Stavanger. Information on physical activity (PA) levels, perceived neighborhood safety, and socio-demography were obtained from questionnaire data collected in the Norwegian county public health survey of Rogaland. Geographic information systems were utilized to compute walkability, vegetation scores and proportion of green space within postcode areas, which subsequently were linked to the survey data. Hierarchical linear regression models were fitted to examine associations between walkability, amount of vegetation, proportion of green space and weekly minutes of PA, and to estimate main and interaction effects of perceived safety on these relationships.

**Results:**

The adults were on average physically active 148.3 min/week. The amount of green vegetation in the neighborhood was positively related to physical activity when adjusting for potential confounders. No such relations were observed for proportion of green space and walkability. Perceived neighborhood safety was significantly related to increased levels of physical activity, but no moderating role of perceived safety was observed.

**Conclusion:**

Although our findings should be interpreted with caution, the results point towards the importance of policymakers, planners, and public health professionals to advocate for safe environments with green vegetation for physical activity in the neighborhood.

## Background

Evidence has proven that daily physical activity reduces the risk of non-communicable diseases (NCDs), such as cardiovascular disease, diabetes, and cancer, as well as depression and anxiety [1–3]. The effects of physical activity are also found to extend beyond health by strengthening general well-being [4]. Despite these renowned and substantial benefits, physical inactivity is still the fourth leading cause of death worldwide [5, 6]. It has been estimated that 27.5% of the world’s population is not sufficiently physically active [7]. This also applies to the Norwegian population, where the inactivity levels remain high with a staggering 68% of adults not meeting the physical activity recommendations [8]. Identifying solutions that can help reverse the physical inactivity trend is therefore a highly prioritized task among public health researchers, practitioners, and policymakers.

In 2018, the World Health Organization (WHO) launched the Global Action Plan on Physical Activity 2018–2030 (GAPPA) to combat physical inactivity as a major public health issue. This action plan sets a global target to reduce physical inactivity by 15% by 2030, and it engages multiple sectors, strategies, and partners to reach its goals [9]. Importantly, many countries worldwide have made effort to incorporate similar measures within their public health plans [5], including Norway. In addition, the Norwegian government has developed a national action plan corresponding to GAPPA that aims to reduce the inactivity levels of Norwegian citizens by 30% by 2030. This aim is intended to be achieved by creating a more activity-friendly society where everyone, independent of age, gender, functional level, and social background, are given the opportunity to live an active life [10]. Neighborhoods are commonly considered as key settings in that regard as they provide infrastructure for various forms of activity through the interaction between the built and the social environment [11–13].

Research carried out over the past two decades has identified built and social characteristics of neighborhoods that seem to promote an active lifestyle among adults [14–17]. This paper draws attention to green space, walkability, and perceived safety. Neighborhood green spaces are considered important arenas for facilitating different forms of physical activity [12, 18–21], and studies have reported that residents living in greener neighborhoods are more likely to walk and participate in physical activity compared to those living in less green environments [22, 23]. Moreover, access to parks have been found to be positively related to moderate to vigorous physical activity (MVPA) and active transport [16, 24]. In a study including 12 countries across five continents, access to neighborhood parks was associated with total levels of physical activity, as well as walking for transport and leisure [17]. Along with green spaces, neighborhood walkability is also found to be conducive to physical activity by promoting walking for various purposes [14, 16, 25, 26]. Living in walkable areas has been linked to more frequent walking for transport and leisure, as well as higher levels of general physical activity in adults [27–29].

Although relationships between neighborhood green space, walkability and physical activity have been extensively studied, important knowledge gaps remain to be filled. First, results of associations between green space and physical activity are inconsistent [18, 30]. While many studies have found positive associations (see e.g., Sallis et al. [24], Sallis et al. [31], and Astell-Burt et al. [32]), there are studies that do not identify any such relationships [33], and some have even found negative associations between access to green space and physical activity levels in adults [34]. Thus, more evidence is needed on how these relationships vary by context and population groups [18], and we have rather limited knowledge on the role of green space and walkability for physical activity among adults within the Nordic region [35–38]. In recently published reviews on the topic [36–38], only five studies from the Nordic context have been identified, of which two studies were conducted in Finland [39, 40], two in Sweden [41, 42] and one in Norway [43]. Anyhow, the Norwegian study examined if new bicycle infrastructure resulted in changes of route among bicyclists and did not assess green space characteristics and walkability [43]. This underscores the need for examining relations between neighborhood green space, walkability, and physical activity in Norway.

Secondly, where most studies have examined whether associations exist between green space, walkability and physical activity, only limited research has investigated potential moderators of such relations [44]. There is much to be learned about how green space and walkability interact with the neighborhood social environment to support physical activity, and one suggested social factor of relevance is perceived safety [44, 45]. Studies have found that residents feeling safe in their neighborhood achieve higher levels of physical activity [46], whereas neighborhoods perceived as unsafe are found to impede walking [47]. The few studies that have examined safety as a moderator of built environment associations with physical activity in adults report somewhat mixed and elusive results [23, 48, 49]. Apart from a Swedish study [23], a possible moderation effect of perceived safety on associations between green space, walkability and physical activity has not been thoroughly investigated in the Nordic context. The results reported by Weimann et al. [23] suggest that perceiving the neighborhood as safe is a prerequisite for the activity promoting effect of green spaces. This may also apply to other built environment characteristics, such as walkability. Thus, we aimed to (i) investigate relationships between neighborhood green space, walkability, and physical activity in Norwegian adults, and (ii) examine whether any such relationships were moderated by perceived neighborhood safety.

## Methods

### Design and data sources

The present study was carried out as part of the project *NORDGREEN - Smart Planning for Healthy and Green Nordic Cites*. The project is collaborating with six cities across the Nordic region, and one of these cities is Stavanger in Norway [50], which we are focusing on herein. Stavanger is a city and a municipality located in Rogaland county in the southwestern part of Norway. With close to 145 000 inhabitants, Stavanger is the fourth largest city in the country [51]. Stavanger municipality consists of nine districts and has a total land area of 241 km^2^.

To address the study aims, we applied a cross-sectional design in which data obtained from the Norwegian County Public Health (NCPH) survey in Rogaland were linked to objectively measured data on neighborhood greenness and walkability within the participants’ postal code areas using geographical information systems (GIS). Details about the NCPH survey have been published elsewhere [52].

### Study sample

The Norwegian Institute of Public Health was responsible for the NCPH survey and the data collection. A random sample of 90 215 adults aged ≥ 18 years living in Rogaland county was drawn from the census register. People reserved from participating, deceased, and those with unverified contact information were excluded, resulting in 77 889 residents being invited to participate by SMS and email. A total of 35 191 (45.2%) adults responded to the survey between September 14 to October 5 in 2020 [52]. From this sample of respondents, we obtained data for all the participating adults living in Stavanger municipality (n = 7057). Two of the districts in Stavanger, Rennesøy and Finnøy, are islands. As these islands mainly consist of rural and agricultural areas with few inhabitants, participants living on these two islands were excluded for comparison reasons. This left us with a total sample size of 5670 adults residing in the urban districts of Stavanger.

### Variables derived from the NCPH survey

Weekly physical activity levels were measured based on two questions capturing frequency and duration of physical activity. Frequency was elicited through the following question: “How often do you work out or exercise in your free time?” The selectable six options were “never”, “less than once a week”, “once a week”, “2–3 times/week”, “4–5 times/week” or “approximately every day”. Duration was assessed with the question “For how long do you usually exercise?” The responses were “less than 15 minutes”, “15–29 minutes”, “30–60 minutes”, or “more than 1 hour”. Inspired by the Nord-Trøndelag Health Study (HUNT2) [53], the two categorical variables were transformed into a continuous variable capturing weekly minutes of physical activity. First, the frequency responses were recoded in the following way: 0 (never), 0.5 (less than once a week), 1 (once a week), 2.5 (2–3 times a week), 4.5 (4–5 times a week) and 7 (approximately every day). Then, the duration responses were recoded using the values 8 (< 15 min), 22 (15–29 min), 45 (30–60 min) and 60 (> 1 h). Finally, the recoded frequency and duration values were multiplied to obtain total weekly minutes of physical activity for each participant, ranging from 0 to 420 min. This variable was treated as the outcome variable.

To measure perceived safety, we used a question addressing how safe the participant felt when he/she was out walking in the neighborhood area. The responses were provided on a 10-point scale ranging from 0 to 10 where higher scores indicate higher perceived safety. This continuous variable was treated as a moderator in the regression analyses.

From the NCPH survey, we also obtained data on age, gender, educational level, and perceived financial status to account for potential confounders based on existing research and theoretical knowledge [45, 54]. Age was provided as a categorical variable and recoded into four categories (i.e., 18–29, 30–49, 50–69 and ≥ 70 years). Educational level was recoded into “high school or less”, “university < 4 years” and “university ≥ 4 years”. Perceived financial status had seven selectable options, which were recoded in the following way: “very hard”, “hard” and “quite hard” were merged into “difficult financial status”, “quite easy” and “easy” were categorized as “easy financial status”, whereas “very easy” remained a separate category.

### Measuring greenness and walkability

Geographical information systems software (QGIS 3.16.6) was utilized to compute greenness and walkability within each participant’s postal code area. Vegetation data were downloaded from the Copernicus websites (Copernicus Global Land Service) to compute a measure of green vegetation using the Normalized Difference Vegetation Index (NDVI). NDVI is a commonly used and validated indicator for measuring vegetation in pixels or grids [55, 56]. The principle underlying NDVI is that different surfaces reflect red and near-infrared light in different ways, and healthy green vegetation reflects more near-infrared light compared to non-vegetated surfaces [55]. The satellite data had a 250 × 250 m grid resolution and were produced from the Sentinet-3 satellite. We downloaded six datasets from April 2020 to July 2020 and used the maximum value to obtain data representing the greenest period based on previous research [57]. A mean NDVI-value for each postal code area was computed. The NDVI index range from – 1 (representing e.g., water bodies and ice) to + 1 (representing only green vegetation). There were no negative values in our data, which means that our mean values can range from 0 to 1 with values close to zero indicating mainly rocks and bare soil and scores close to 1 indicating more dense green vegetation in the neighborhood. In addition to the NDVI scores, we computed the total proportion of green space within each postal code area. The operational definition of this measure is presented below.

Several operational definitions of walkability exist within the literature [58], and the index developed by Frank et al. [59] is commonly applied. This walkability index has been developed in the US context and is calculated based on a summary score of intersection density, residential density and land-use mix within a defined geographical area (e.g., a district, neighborhood, or postal code area) [59]. The walkability measure applied in this study is based on the index developed by Frank et al. [59]. However, to account for the Norwegian context and the land-use pattern in Stavanger, which is characterized by high proportions of forest and agricultural land, we computed an index representing a summary score of the proportion of green space, population density and intersection density. A detailed description of the components and how the score was computed is provided below.

Maps and associated data for computing the walkability index were downloaded in November 2021. The *proportion of green space* was computed from data obtained through national land-cover and land-use maps from the Norwegian Mapping Authorities. The proportion of the total land area (in km^2^) devoted to green space (i.e., forests, parks, and cemeteries) was computed within each postal code area. This measure was used as single predictor of weekly minutes of physical activity and as a component of the walkability index. We operationalized and measured *population density* as the total number of residents per km^2^ within each postal code area. The most recent Statistical Grid Dataset (250 × 250 m) with population data from Statistics Norway was utilized. Data for computing *intersection density* was obtained from road maps provided by the Norwegian Mapping Authorities. First, the total number of street intersections with three or more legs were identified within each postal code area. Subsequently, the total number of intersections was divided by the total land area (in km^2^) of each postal code area to obtain a density measure. In line with the computations of Frank et al. [59], the values for each component of the index were normalized using a z-score. Finally, we computed the walkability index by summarizing the z-scores for each postal code area using the following formula: walkability = z(proportion of green space) + z(residential density) + z(intersection density).

### Statistical analysis

Background characteristics of the total sample were analyzed using descriptive statistics, and results from these analyses are presented as frequencies and proportions, as well as mean values with standard deviations. To examine associations between greenness, walkability and weekly minutes of physical activity, hierarchical linear regression analyses were performed. Prior to running these analyses, participants with missing data on all key variables were removed from the dataset, and consequently, 5307 adults were included in the final analytical sample. Three regression models, one for each built environment characteristic against the physical activity outcome, were fitted. The independent variables and interaction terms were added to the models in three consecutive steps. In the first step, weekly minutes of physical activity was regressed on the independent variables NDVI, proportion of green space and walkability, adjusted for age, gender, educational level, and perceived financial status. In the second step, perceived neighborhood safety was entered into the models. Before creating the interaction terms, the variables included in the terms were standardized to avoid possible multicollinearity issues [60]. Finally, the interaction terms of NDVI*perceived safety, proportion of green space*perceived safety, and walkability*perceived safety were added to the models. Unstandardized coefficients (B) with 95% confidence intervals (CI) are reported to present the change in adults’ weekly minutes of physical activity. All statistical analyses were performed using SPSS version 28.0, and p-values < 0.05 were considered statistically significant.

## Results

### Characteristics of the participants

Descriptive statistics for the total sample and other key variables are detailed in Table 1. The sample comprised of 2623 men (46.3%) and 3047 women (53.7%). Most of the participants aged between 30 and 69 years (72.7%), and just above one third were highly educated. On average, the participants were physically active close to 150 min/week, and generally, they perceived their neighborhoods as safe (mean = 8.84, SD = 1.55). The mean walkability score for inhabitants in the urban areas of Stavanger municipality was 0.28 (SD = 1.57), whereas the mean vegetation score (NDVI) was 0.65 (SD = 0.13). The mean proportion of green space, including forests, parks, and cemeteries, was 9.8% (SD = 8.7) (Table 1). As indicated by the range in proportion of green space (0.0-51.4%), there were postal code areas that did not had forest, parks, and cemeteries. However, all participants lived in areas that had some green vegetation (range of the NDVI score = 0.26–0.87).

**Table 1:** Study sample characteristics and descriptive statistics for key variables (n = 5670)

Characteristics	n (%)
Gender	
Male	2623 (46.3)
Female	3047 (53.7)
Age groups	
18–29	967 (17.1)
30–49	2067 (36.5)
50–69	2050 (36.2)
70+	586 (10.3)
Perceived financial status	
Hard	925 (17.2)
Easy	2726 (50.6)
Very easy	1733 (32.2)
Educational level	
High school or less	2214 (39.2)
University < 4 years	1359 (24.0)
University ≥ 4 years	2080 (36.8)
	**Mean (SD)**
Min/week physical activity	148.43 (116.95)
Perceived safety (0–10)	8.84 (1.55)
NDVI (0–1)	0.65 (0.13)
Proportion of green space (%)	9.8 (8.7)
Walkability	0.28 (1.57)

### Greenness, perceived safety, and physical activity

Results of the regression analyses estimating relationships between greenness, perceived safety, and weekly minutes of physical activity among adults in urban areas of Stavanger are shown in Table 2. We found a positive relationship between NDVI and physical activity, adjusted for potential confounders. The maximum effect size was 26.8 min/week (95% CI = 2.1, 54.4) with a one-unit increase (from 0 to 1) in the NDVI score, indicating that 26.8 more minutes of PA can be achieved weekly going form an area characterized by mainly rocks and bare soil to an area with mainly dense green vegetation. In our material, the NDVI scores ranged from 0.26 to 0.87, and the adjusted mean difference in physical activity was 22.4 min/week between people living in the area with the lowest vs. the highest NDVI score. When safety was added to the model (Step 2, model 1), the coefficient decreased, and the observed significant association vanished. However, a positive relationship between perceived safety and physical activity was found, with a one-unit increase in perceived safety resulting in 5.9 (95% CI = 3.8, 8.1) more minutes of weekly physical activity. Adding the interaction term in the final step (Step 3, model 1), revealed no moderation effect of perceived safety on the observed relationship between NDVI and physical activity (Table 2). The regression coefficient for the proportion of neighborhood green space was positive but not significantly related to adults’ physical activity levels (B = 3.7; 95% CI = -31.8, 39.2). As in model 1, a significant positive relationship was found between perceived safety and weekly minutes of physical activity in model 2, but no moderation effect was detected (Table 2).

**Table 2:** Estimated regression coefficients (B) with 95% CI for associations between neighborhood greenness and physical activity

Predictors	Step 1^a^	Step 2^a^			Step 3^a^
*Model 1* NDVI	26.8 (2.1, 51.4)*	20.3 (-4.3, 45.0)			19.9 (-4.9, 44.8)
Perceived safety		5.9 (3.8, 8.1)***			5.9 (3.7, 8.0)***
Interaction term					
NDVI*perceived safety					-0.4 (-3.2, 2.4)
Adjusted R Square	0.159	0.175			0.175
*Model 2* Proportion of green space	3.7 (-31.8, 39.2)		5.6 (-29.8, 41.0)	117 (-47.8, 282.5)	
Perceived safety			6.1 (4.0, 8.2)***	6.2 (4.1, 8.2)***	
Interaction term					
Proportion of green space*perceived safety				-1.1 (-2.8, 0.5)	
Adjusted R Square	0.156		0.174	0.175	

Abbreviations: CI, confidence interval, *p < 0.05, **p < 0.01, ***p < 0.001

^a^ Adjusted for age, gender, educational level and financial status

### Walkability, perceived safety, and physical activity

Associations between walkability, perceived safety and physical activity are presented in Table 3. A weak, negative relationship was observed between walkability and physical activity levels in our sample of Norwegian adults, but the result was not statistically significant (B = -1.4; 95% CI = -3.3, 0.6). As for the model presented in Table 2, the regression coefficient slightly changed when entering perceived safety into the model (Step 2, Table 3). No moderation effect of perceived safety on the relationship between walkability and physical activity was found, but perceived safety was positively related to weekly minutes of physical activity also in this model (Table 3).

**Table 3:** Estimated regression coefficients (B) with 95% CI for associations between neighborhood walkability and physical activity

Predictors	Step 1^a^	Step 2^a^	Step 3^a^
*Model 3* Walkability	-1.4 (-3.3, 0.6)	-1.0 (-3.0, 1.0)	-1.0 (-3.0, 1.0)
Perceived safety		6.0 (3.9, 8.2)***	6.0 (3.9, 8.2)***
Interaction term			
Walkability*perceived safety			-0.34 (-3.3, 2.6)
Adjusted R Square	0.159	0.174	0.174

Abbreviations: CI, confidence interval, *p < 0.05, **p < 0.01, ***p < 0.001

^a^ Adjusted for age, gender, educational level and financial status

## Discussion

This cross-sectional study of Norwegian adults showed that living in greener neighborhoods was associated with increased levels of physical activity. No such relationships were found for the overall proportion of forests, parks, and cemeteries, and walkability. Perceived safety did not seem to act as a moderator for the associations under study but perceiving the neighborhood area as safer was related to more minutes of physical activity per week. The positive relationship identified between neighborhood greenness and physical activity aligns with results of previous research [17, 22, 23]. Although the association was statistically significant after adjusting for age, gender, and socioeconomic variables, it should be stated that the maximum effect size was 26.8 min/week, and all participants lived in areas that had some green vegetation. Hence, the variance in NDVI scores between the participants is quite small, indicating that the estimates were less substantial. Moreover, the confidence interval for the regression coefficient was wide.

Only one of the green space measures computed in this study was found to be positively related to the adult citizens’ physical activity levels. A reason for this might be the construct validity of the green space measures applied in relation to physical activity, as well as the nature of the physical activity measure. To assess the total proportion of green space in each postal code area, we included forests, parks, and cemeteries, which are commonly used for physical activity purposes [61, 62] However, rather than assessing domain specific physical activity (e.g., time spent on physical activity in green spaces), we measured weekly minutes of overall physical activity, which is accumulated in a variety of settings both indoors and outdoors, such as garden, streets, and squares with vegetation. These outdoor spaces are examples of settings for physical activity that are not captured through the green space types included in the proportion measure applied. The NDVI score, on the other hand, captures all green vegetation identified in an area, including gardens, streets, and open squares [55]. This might explain why we found a significant association between the amount of green vegetation and overall physical activity and not for the total proportion of neighborhood green space.

Contrary to our results on the amount of green vegetation in the neighborhood, we found a negative association between walkability and weekly minutes of physical activity. This is inconsistent with previous studies [27–29, 63], but our estimate was weak and not statistically significant. A reason for this lack of clear findings for walkability could be how physical activity was defined and measured in the present study. The relationship between neighborhood walkability and physical activity may differ depending on the physical activity construct or measure used, such as general physical activity, walking, or other forms of active transport. Research has demonstrated walkability features to be more positively related to active transport than overall physical activity [16].

As discussed previously, we used a measure of overall physical activity, which capture activity irrespective of context. Even though outdoor walking is reported as the most common way to be physically active in the Norwegian adult population, surveys show that indoor activities like exercising in the gym and swimming are popular [64]. Such activity might only be affected by walkability to a limited extent e.g., if people are using active transport to these places. Another explanation to our finding is that high neighborhood walkability does not imply that residents perceive the area as walkable. The walkability index is a simplified measure consisting of a few selected built environment features. Other factors like traffic, quality of sidewalks, lighting, and noise could influence how walkable a neighborhood is perceived. For instance, densely built neighborhoods usually have more facilities but also more traffic [65], making it less pleasant to walk or bike. Nevertheless, these factors were not considered in this study.

Based on previous studies linking built environment characteristics to higher levels of physical activity among inhabitants perceiving their neighborhoods as safe [23, 48, 66, 67], we wanted to examine perceived safety as a moderator within the Norwegian context. We hypothesized that feelings of unsafety could be a barrier for staying outside, whereas higher safety could encourage physical activity in the neighborhood, and thereby, play a role in relations between greenness, walkability, and physical activity. However, this hypothesis was not supported by our results, and our findings add to the inconsistent empirical evidence of the moderator role of safety in such relationships [14, 68, 69]. Overall, our respondents perceived their neighborhoods as safe, with an average score of 8.84 (SD = 1.55). This mean score in our sample suggests lack of variability causing a potential ceiling effect. This may limit our ability to detect significant interactions and could have contributed to an underestimation of the role of perceived safety in relations between neighborhood greenness, walkability, and physical activity. As such, a more detailed measure might be required to assess perceived safety more adequately.

Although no interaction effects were identified, a notable result in this study was the positive association observed between perceived safety and the adults’ physical activity levels. The regression analyses showed that a one-unit increase in perceived safety contributed to about 6 more minutes of weekly physical activity. Similar results have been described in several other studies [65–67]. For example, Foster et al. [66] reported an 18-minutes increase in weekly physical activity using a five-point Likert scale. The work reported in the present study provides support for future studies to explore the role of perceived safety for physical activity within the Nordic countries, and to understand more about the factors contributing to perceptions of high neighborhood safety.

### Strengths and limitations

The large sample of inhabitants from the city of Stavanger represents an important strength of this study as it increased the likelihood of representativeness. Importantly, the response rate in the NCPH in Rogaland was high compared to similar surveys in Norway [52]. Nevertheless, with a response rate of 45.2% we cannot rule out selection bias. Use of GIS-measures for assessing greenness and walkability is considered a methodological strength as it eliminated the risk of single source bias influencing associations between the environmental characteristics and adults’ physical activity. Yet, we relied on self-reports of frequency and duration of physical activity, which are prone to both recall bias and social-desirability bias.

When assessing physical activity, the aim is usually to identify duration, frequency, intensity, and type of activity performed [70]. Information on type of activity was not available and intensity was not considered, which is a weakness in this study. However, the questions used to assess physical activity have been validated against objective measures and the International Physical Activity Questionnaire. Moreover, the recoding of the variables was based on previous research from Norway [71]. Yet, it should be stated that the two categorical variables that were recoded are prone to desirability bias, which in turn has an impact on the computed weekly minutes of physical activity. The validity of the conversion is also limited investigated. The participants in our study reported close to the WHO’s recommended level of 150 min of weekly physical activity [72], with an average of 148.3 min/week. Considering that our variable did not account for intensity, which is included in the WHO recommendation, these numbers are not entirely comparable. Still, the average value shows that the respondents were highly active on a regular basis. As only 30% of Norwegian adults are found to reach the recommended physical activity levels [10], the representativeness of our sample could be questioned. Another crucial limitation of the physical activity measure was that the context was not considered. This likely restricted us from identifying clear associations between the built environment features and physical activity in this sample of Norwegian adults.

We used postal code areas for defining neighborhoods and computing GIS-variables. As such, there is uncertainty in exposure to the built environment, which introduces measurement errors. It is reasonable to assume that the regression estimates are influenced by how neighborhoods were defined as we faced the “uncertain geographic context problem” [73]. Recent studies have explored how associations vary according to how neighborhoods are delineated [74, 75]. Both Laatikainen et al. [74] and Zhao et al. [75] showed that different spatial units of analysis yielded different estimates of relations between the built environment and health outcomes. Due to data protection and ethics, we had to use postal code areas and were not able to explore other spatial units. This likely limited our ability to detect associations between the greenness, walkability, and physical activity as administrative units (i.e., postal code areas) do not capture the dynamic nature of everyday human behaviour [74, 75]. Regardless of that, we used a validated metric to capture neighborhood greenness [55]. The strength of using NDVI, in comparison to land-cover and land-use maps, is that it includes all green vegetation (e.g., private gardens, parks, cemeteries, golf courses, forests, and green corridors in the urban landscape), which can be suitable arenas for physical activity [18]. However, NDVI does not provide any information concerning the qualities of green spaces. Size and factors like amenities and maintenance seem to be important for use of green spaces and related health benefits [15, 45, 76]. As an objective measure, NDVI also has another weakness. In a Dutch [77] and Norwegian study [78], perceptions of green space were found to be a stronger predictor for usage than objective measures, such as NDVI. Understanding how and why residents interact with neighborhood green spaces appears to be important for planning processes. Thus, qualities of green spaces and how it influences use and public health outcomes should be more thoroughly investigated in the Norwegian and Nordic context [58]. Based on the walkability index of Frank et al. [59], we created a composite score that was adapted to the Norwegian context and land-use pattern. This could be considered both a strength and a weakness, as the applied measure has not been validated in Norway.

Finally, this study is limited by the constraints of the cross-sectional design, which prevents us from making inferences about causality. Although we adjusted for important confounders in the analyses, other variables not available through the NCPH survey could influence the results. This includes, amongst others, motivation for physical activity, traffic, noise, and maintenance of the built environment.

### Implications and concluding remarks

Urban residents need neighborhoods that support an active lifestyle. Hence, knowledge on whether and how the built and social environment can support physical activity is important for planners and policymakers aiming to create an activity-friendly society [10]. In conclusion, we found that living in neighborhoods with more green vegetation and perceiving the area as safer were positively associated with physical activity among adults in urban areas of Stavanger, Norway. The same association was not observed for proportion of green space and walkability in our study sample. These findings suggest that providing green vegetation and fostering safety in neighborhoods could be essential to support physical activity. This knowledge can guide and inspire planning and design of neighborhoods from a public health perspective. We also believe that the results add evidence-based strength to present policies that, among others, emphasize safety and access to green space as important health-promoting neighborhood characteristics. Lastly, future studies should aim to further investigate these relations and scrutinize pathways through which the built and social environment influence physical activity using more refined methods to overcome the limitations of this present study. Particularly, there is a need for more longitudinal studies addressing these associations in the Nordic context.

## Data Availability

The data that support the findings of this study are available from the Norwegian Institute of Public Health, but restrictions apply to the availability due to agreements and approvals involving data security for participants from the Norwegian Data Protection Authority. Data are however available from the authors upon reasonable request and with permission of the data owner if requestors wish to access the data for the purposes of checking analyses. Any requests should be directed to the corresponding author Emma Charlott A. Nordbø.

## List of abbreviations

PA: physical activity
MVPA: moderate-to-vigorous physical activity
WHO: World Health Organization
GAPPA: Global Action Plan on Physical Activity
NCPH: Norwegian County Public Health survey GIS:geographical information systems
CI: confidence interval
NDVI: Normalized Difference Vegetation Index.
